# Editorial: Intranasal Drug Delivery: Challenges and Opportunities

**DOI:** 10.3389/fphar.2022.868986

**Published:** 2022-03-02

**Authors:** Ana Fortuna, Katharina Schindowski, Fabio Sonvico

**Affiliations:** ^1^ Faculty of Pharmacy, University of Coimbra, Coimbra, Portugal; ^2^ Coimbra Institute for Biomedical Imaging and Translational Research (CIBIT), Coimbra, Portugal; ^3^ Institute for Applied Biotechnology, University of Applied Sciences Biberach, Biberach an der Riss, Germany; ^4^ Department of Food and Drug, University of Parma, Parma, Italy; ^5^ University Research Centre for the Innovation of Health Products, Biopharmanet-TEC, University of Parma, Parma, Italy

**Keywords:** intranasal, nose to brain, systemic delivery, nasal epithelia, nasal formulations

For a long time, intranasal administration is ascribed as an effective route to deliver drugs into the systemic circulation, resulting in rapid onset and higher drug bioavailability than classical extravascular administration routes. More recently, intranasal administration started to gain attention as a potential delivery route to the brain due to the unique connection between the central nervous system and the exterior environment, which is made by the olfactory nasal neuroepithelium. For instance, esketamine nasal spray (Spravato^®^) was recently approved as a new fast-acting antidepressant drug for the therapy of treatment-resistant depression and major depression with suicide ideation.

Indeed, the nasal route is highly suitable for minimally-invasive drug delivery as the airway mucosa presents a good permeability and efficient absorption (Gänger and Schindowski, 2018). The human nasal mucosa has an impressively large surface area and turbinates humidify, warm and filter the inspired air. Nasal secretions and inhaled particles are transported *via* mucociliary clearance to the nasopharynx where they are swallowed or expectorated. Importantly, the nasal mucosa provides a very relevant immune function since countless inhaled pathogens are filtered here and transported to the nose-associated lymphoid tissue (NALT) (Pabst, 2015). Therefore, intranasal vaccinations become more and more attractive as a needle-free form of vaccinations.

Both systemic and brain-delivery through nasal administration have been (and remain) challenging, not only because of anatomic, physiological and histological characteristics of the nasal cavity but also due to the underlying mechanisms of drug absorption and drug delivery through nasal epithelium.

The nasal respiratory epithelium covers up to 90% of the nasal cavity in humans and up to 50% in rodents and is highly vascularized, hence well suitable for the systemic absorption of drugs. The human olfactory cleft at the roof of the nasal cavity down to the superior parts of the turbinates are covered with olfactory mucosa that covers about 50% of the nasal cavity in rodents and approximately 10% in humans. Olfactory sensory neurons have their cell bodies located in a peripheral epithelium. Interestingly, the olfactory nerve bundles appear to have an impact in nose to brain drug delivery (Ladel et al., 2018; Maigler et al., 2021).

Therefore, the region where the drug is applied in the nose is critically important and can direct the drug via different pathways to distinct locations (see Table 1).

**TABLE 1:** Overview of drug delivery pathways related to the nasal cavity for different drugs.

Drug delivery pathways related to the nasal cavity		Clinical examples
Local administration	Respiratory mucosa	decongestants, antiviral drugs Higgins et al. (2020)
Systemic delivery	Respiratory mucosa	calcitonin, sumatriptan, desmopressin
Immunotherapy	Respiratory mucosa/NALT	seasonal flu, polyclonal IgGs Vonarburg et al. (2019)
CNS delivery	Olfactory mucosa	oxytocin, insulin

Spindler et al. and the group of Otmar Schmid (Farnoud et al.) focused therefore on the olfactory region for CNS drug delivery. Zheng and Kendrick review the nose-to-brain delivery of oxytocin while Dalvi et al. present the CNS-uptake *via* thermoresponsive gel formulation. Finally, Flamm et al. established a technique to target selectively either the olfactory or the respiratory region in mice, hence, improving, the quality of future intranasal *in vivo* studies.

Thus, intranasal drug delivery and consequent targeted organs depend on nasal cavity and epithelia conditions as aforementioned but also on formulations type. In fact, while traditional nasal formulations on the market are relatively simple solution of suspension, there is growing evidence that the use of new advanced approaches are needed to foster the next generation of nasal products (Spindler et al., Forbes et al., 2020). In this regard, nanomedicines appear as a valid approach, both as liquid and powder formulation, to overcome some of the limitations associated with nasal administration route and enable the administration of drug for systemic (Farnoud et al., Fortuna et al., 2014) or nose-to-brain delivery (Zheng and Kendrick, Bicker et al., 2020) and antigens and/or adjuvants for mucosal vaccination (Dalvi et al., Bernocchi et al., 2017). Indeed, nanocarriers can provide several desirable features suitable for an optimized nasal administration, such as increased solubility, protection from chemical and enzymatic degradation, mucoadhesion or mucopenetration leading to prolonged retention in the nasal cavity, control of drug release kinetics, enhancement of absorption with consequent improved bioavailabitly, reduction of side effects (Flamm et al., Clementino et al., 2021). This however has to be achieved through the careful selection of bioactive excipients able to provide the nanocarriers with the physical and biopharmaceutical properties more adapted to the specific application. Among the excipients available, lipids and polysaccharides have been demonstrated, on the top of excellent biocompatibility, to have remarkable biological effects. Dalvi et al. proposed rufinamide-loaded chitosan nanoparticle for the direct nose-to-brain delivery demonstrating a further improvement in direct transport efficiency (DTE%) and direct transport percentage (DTP%) to the CNS when nanoparticles are embedded in a thermogelling xyloglucan formulation (Dalvi et al.). This results could also be attributed to the mucoadhesive and penetration enhancing properties provided by chitosan (Casettari and Illum, 2014). Spindler et al. proposed PLGA nanoparticles embedded in chitosan microparticles as a nasal powder formulation, evidencing an accelerated uptake in porcine olfactory mucosa compared to simple PLGA nanoparticles driven by the combination of enhanced paracellular, transcellular and intraneuronal transport. The group of Fabio Sonvico, on the other hand, demonstrated that hybrid lecithin-chitosan nanoparticles display an enzyme triggered release of the encapsulated simvastatin in presence of antibacterial enzymes present in nasal secretions, potentially enabling statin pleiotropic effects for neurodegenerative diseases treatment (Clementino et al.). Finally, Silva and co-authors investigated the co-administration of the antidepressant escitalopram and paroxetine using nanostructured lipid carriers (NLC). It was evidenced that enhanced nose-to-brain transport was achieved for more lipophilic drug, i.e., paroxetine, in particular for NLC modified with the natural bioactive compound borneol (Silva et al.).

The widespread investigations compiled in this special issue clearly corroborate that intranasal delivery is an opportunity to treat currently incurable diseases. However, to accurately screen, optimize and develop successful therapies, *in silico, ex vivo, in vitro* and *in vivo* methodologies must be carefully applied as demonstrated by Silva et al.
